# A single-dose, open-label, randomized, scintigraphic study to investigate the gastrointestinal behavior of 2 triple-combination cold products (acetaminophen, phenylephrine, and dextromethorphan) in healthy male volunteers

**DOI:** 10.1186/s13063-022-06037-x

**Published:** 2022-02-08

**Authors:** Pascal Mallefet, Marianna Armogida, Walter J. Doll, Richard C. Page, Erik P. Sandefer

**Affiliations:** 1 GSK Consumer Healthcare, Route de l’Etraz 2, 1260 Nyon, Switzerland; 2 Scintipharma, Inc., Lexington, KY USA

**Keywords:** Common cold, Gastric emptying, Radionuclide imaging, Powders, Beverages, Duodenum, Gamma scintigraphy

## Abstract

**Background:**

Common cold symptoms may be mitigated by products in caplet, nasal spray, and oral solution formulations, although variations exist in the bioavailability of the active ingredients contained within these products. Rapid gastric emptying (GE) of these active ingredients is important for reducing the delay between drug absorption and onset of cold symptom relief. Hot drink cold remedies are associated with greater comfort and may enhance the bioavailability of active ingredients. The objective of this study was to characterize the gastrointestinal transit of powder (reconstituted in hot water) and caplet formulations of commercially available multisymptom cold medications.

**Methods:**

This was an open-label, single-dose, parallel-group study. Healthy male adults under fasted conditions were randomized 1:1 to receive a single dose of radiolabeled Theraflu Daytime Severe Cold and Cough powder for oral solution or radiolabeled Theraflu ExpressMax Daytime Severe Cold and Cough caplet. External gamma scintigraphy was utilized to monitor GE and intestinal transit of two radiolabeled drug formulations.

**Results:**

A total of 28 participants completed the study. The mean ± SE GE onset times were 1.1 ± 0.3 min and 8.5 ± 1.8 min for powder and caplet formulations, respectively. The mean ± SE GE completion times were 121 ± 13 min and 65 ± 13 min, respectively. Despite the similar mean times to GE25%, the powder had later mean GE50% (23 ± 3.0 vs 16 ± 3.2 min, respectively) and GE90% (85 ± 12 vs 36 ± 9 min, respectively) than caplets. Caplets had a shorter overall GE half-life, lower total gastric exposure, and faster transit time through the small intestine versus the powder formulation. No serious safety events were observed.

**Conclusion:**

The results of this study in healthy male adults suggest that the Theraflu powder formulation had a more rapid GE onset but longer time to GE completion than the caplet formulation.

**Trial registration:**

ClinicalTrials.gov NCT03415243

**Supplementary Information:**

The online version contains supplementary material available at 10.1186/s13063-022-06037-x.

## Background

The common cold is the most frequent acute illness found throughout the industrialized world and is associated with a high economic burden through lost productivity and expenditures for treatment [1–3]. On average, adults experience 2–4 colds per year, yet despite this prevalence, there is currently no cure and only symptomatic relief treatment, although the impact of symptoms can be mitigated by several prescription medicines and over-the-counter products [4, 5]. Cold remedies and multisymptom drug combinations are available in a variety of formulations, including caplets, nasal sprays, and oral solutions. Hot drink remedies are associated with greater subject comfort and provide active ingredients in solution, which may result in the active ingredients reaching the bloodstream faster and being bioavailable more quickly compared with tablet formulations [6, 7].

Theraflu Daytime Severe Cold and Cough powder for oral solution and Theraflu ExpressMax Daytime Severe Cold and Cough caplets (both GSK Consumer Healthcare) are among the numerous commercially available multisymptom formulations [8, 9]. Both products contain the same dosage of active ingredients: acetaminophen, phenylephrine, and dextromethorphan; however, ExpressMax caplets contain an additive to provide a buccal warming sensation when placed in the mouth. The powder for oral solution product is available in sachet for reconstitution with hot water [8]. The active ingredients pass through the stomach unabsorbed. Once they reach the duodenum, absorption is rapid and significant; therefore, rapid gastric emptying (GE) is important for reducing the delay between drug ingestion and the onset of symptom control [10–12].

This clinical study was designed to characterize the gastrointestinal (GI) transit of powder (liquid) and caplet (solid) formulations of these commercially available multisymptom formulations using gamma scintigraphy. We assessed and quantified GI transit of radiolabeled drug formulations in healthy male adults and evaluated the safety of these products.

## Methods

### Participants

For study eligibility, candidates were required to be healthy adult males aged 21–45 years, with a body mass index ranging from 17.5–30.5 kg/m^2^ and total body weight > 110 lb (> 50 kg). Advanced aging induces morphological and functional changes to the GI tract that can affect transit time, metabolism, and gut permeability, potentially compromising effective drug absorption in an elderly population [13]. Thus, the age range in this population of healthy young men was chosen to minimize variables such as metabolism and gut permeability that can affect GI transit time and, in an older population, compromise effective drug absorption. Females were excluded from participation, as the menstrual cycle has been associated with alterations in GE patterns [14]. Participants were also excluded if they followed a vegetarian diet, due to evidence that acetaminophen absorption [15] and/or metabolism [16] may be impaired in this subpopulation. Other exclusion criteria included cumulative therapeutic or diagnostic radiation exposure > 150 millisieverts above background levels in the last 12 months; evidence or history of clinically significant laboratory abnormalities or clinical disease within the last 5 years that may increase risk (e.g., renal, endocrine, or psychiatric disease); drugs and dietary supplements with a potential impact on the study results (as determined by the investigator) within 14 days or 5 half-lives, whichever was longer, prior to the first dose of the investigational product; and/or recent consumption of grapefruit or related citrus fruits 14 days prior to the first dose of investigational product.

### Study design

**Fig. 1:**
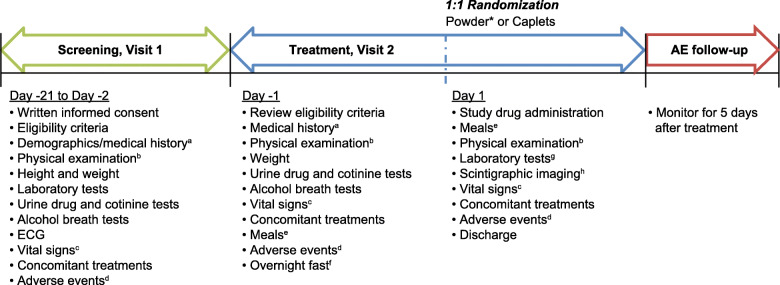
Study design. *Powder dissolved in hot water. ^a^Medical history recorded was any existing or resolved condition that started prior to informed consent. Changes in the medical history were assessed at visit 2 (day 1). ^b^Full physical examination occurred on visit 1 screening and on visit 2 (day − 1). A brief physical examination occurred on visit 2 (day 1) before randomization and prior to discharge. ^c^Vital signs performed at visit 1 and visit 2 (day − 1) included BP, RR, PR, and oral temperature. At visit 2 (day 1), vital signs included BP and PR performed before randomization and after the last scintigraphic image was obtained. ^d^AEs (serious and non-serious) were collected from the time the subject signed the informed consent form until 5 days following the last administration of the investigational product. ^e^Subjects were given a standard lunch 4 h post-dose and a standard dinner 10 h post-dose. ^f^Fasting occurred from 10 h prior until 4 h after study drug administration. ^g^Laboratory tests on visit 2 (day 1) were conducted after the final scintigraphic image was taken, prior to dinner. ^e^Scintigraphic acquisitions were taken beginning after dose administration until 10 h post-dose. AEs, adverse events; BP, blood pressure; ECG, electrocardiogram; PR, pulse rate, RR, respiratory rate

This was an open-label, randomized, single-dose, parallel-group study (Fig. 1) conducted on March 1–29, 2018, at a single study center (Scintipharma, Inc., Lexington, KY, USA; ClinicalTrials.gov identifier NCT03415243; https://www.clinicaltrials.gov/ct2/show/NCT03415243). The study protocol was approved by an institutional review board (Advarra IRB, Columbia, MD, USA) prior to the administration of any study drug.

This study consisted of 2 periods: period 1 was the screening visit occurring between days − 21 to − 2, and period 2 consisted of day − 1 in which patients received a final screening for inclusion and day 1 in which patients received study treatment and assessments. During screening visit 1, written informed consent, demographics, and medical history were obtained, and urine drug and alcohol breath tests were performed. On day − 1 of the treatment visit, final eligibility was ascertained and confirmed, and subjects were admitted to the testing unit. On treatment visit day 1, subjects were randomized and received treatment and assessments. The evening before the treatment visit, participants were admitted to the research unit to ensure compliance with fasting conditions and received a standardized evening meal comprising an 8-oz portion of grilled tenderloin tips, a baked potato with butter and sour cream, salad with dressing, green beans, roll, and a decaffeinated 12-oz soda for a total calorie count of 1054 kcal (383 kcal from fat). Participants were required to fast (nothing to eat or drink except room-temperature noncarbonated water) from 10 h prior until 4 h after study drug administration (water was permitted until 1 h before the study product was dispensed). During the pretreatment phase, the eligibility criteria were reviewed, and medical history and urine drug/alcohol breath tests were performed again. On the day of treatment (visit 2, day 1), the study drug was administered and 2 standard meals were provided 4 and 10 h post-dose; lunch consisted of a half-pound cheeseburger (with lettuce, onion, tomato, pickles, mustard, and ketchup), a 10-oz bag of potato chips, 2 oatmeal cookies, a 4-oz fruit cup, and a 12-oz decaffeinated soda for a total calorie (kcal) count of 1144 (464 calories [kcal] from fat), while dinner consisted of a grilled chicken breast (whole), a baked potato (with butter and sour cream), salad (with dressing), a roll, and a 12-oz decaffeinated soda for a total calorie (kcal) count of 1027 (275 calories [kcal] from fat). Water was also permitted at the 4-h post-dose luncheon meal.

Study endpoints, including scintigraphic imaging, were assessed throughout the day. After the last scintigraphic imaging was performed, blood samples for laboratory testing were taken, a brief physical examination was performed, and participants were discharged. Participants were monitored for potential safety events for a subsequent 5 days after study drug administration.

### Study treatment

Participants were enrolled by the study investigator and randomized 1:1 to receive either a single dose of Theraflu Daytime Severe Cold and Cough powder for oral solution (“powder formulation”) or a single dose of Theraflu ExpressMax Daytime Severe Cold and Cough caplets (“caplet formulation”) under fasted conditions. Treatments were administered to participants in an open-label manner by the investigator, using a computer-generated, sequentially numbered randomization list with a block size of 4 provided by a third-party vendor (Syneos Health, Morrisville, NC, USA) and approved by the study sponsor. Each randomization number was assigned to either treatment A or treatment B in blocks; the first 4 allocations were B, B, A, and A; the next 4 were A, A, B, and B; and the next were B, A, B, and A, and so forth.

For the powder formulation, 1 sachet of powder for oral solution (containing acetaminophen 650 mg + phenylephrine 10 mg + dextromethorphan 20 mg) was reconstituted with 225 mL of hot (not boiling) water. A small volume (1–10 μL) of the radioactive marker technetium-99m diethylenetriaminepentaacetic acid (^99m^Tc DTPA, 108 μCi) was added to the reconstituted powder solution after the oral solution cooled to between 40 and 50 °C. Participants randomized to the powder formulation ingested this oral solution when the temperature was between 35 and 45 °C. The caplet formulation consisted of 2 caplets (each containing acetaminophen 325 mg + phenylephrine 5 mg + dextromethorphan 10 mg), each radiolabeled with 54 μCi ^99m^Tc DTPA. Caplets were taken with 225 mL of room temperature noncarbonated water to approximate the total volume of the reconstituted powder formulation administered in the comparator group.

### Assessment technique

External gamma scintigraphy (Scintipharma, Inc. [Lexington, KY, USA]) with ^99m^Tc DTPA was utilized to monitor GE and intestinal transit of the radiolabeled drug formulations, as this technique has been used extensively as a non-invasive approach to assess the in vivo transit performance of drug delivery systems. External gamma scintigraphy is considered the gold standard marker for GE, as it provides information on deposition, dispersion, location, and movement of radiolabeled dosage forms [17, 18]. Although scintigraphy requires radiation, the dose of ^99m^Tc DTPA is considered to be minimal [19]. All participants received a maximum individual ^99m^Tc DTPA dose of 108 μCi (4 megabecquerel). As ^99m^Tc DTPA has a physical half-life of 6 h [20], scintigraphy data were corrected for decay prior to calculation of these GE variables.

Scintigraphic acquisitions were taken after dose administration until 10 h post-dose. Subjects underwent a series of consecutive anterior scintigraphic images, of 60-s duration each on day 1 of each period at 0–15 min (continuous) and at 20, 25, 30, 35, 40, and 45 min, and 1, 1.25, 1.5, 1.75, 2, 2.25, 2.5, 2.75, 3, 3.25, 3.5, 3.75, 4, 4.25, 4.5, 4.75, 5, 5.5, 6, 7, 8, 9, and 10 h. A window of ± 2 min was allowed for each imaging period up to 1 h (inclusive) and ± 3 min after 1 h.

### Analyses

The primary analyses were time to onset of GE (time to reach duodenum) and time to completion of GE (when 0% of the drug remained in the stomach). Additional scintigraphy variables included time to GE25%, GE50%, and GE90%; GE terminal half-life (t_1/2_); amount of product remaining in the stomach at 15, 30, 45, 60, 75, 90, 105, 120, 180, and 240 min post-dose; small intestinal transit times; and area under the GE curve (AUC). The small intestinal transit times were calculated as the time for 50% of the radioactive marker to move through the small intestine, which is the difference between the time for 50% of the marker to arrive at the cecum/colon region less the time for 50% of the marker to empty from the stomach (as assessed via scintigraphy images).

### Safety

Safety was monitored in the safety population via documenting adverse events (AEs), serious adverse events (SAEs), and changes in physical examinations, laboratory tests, vital signs, and electrocardiogram results. Study participants were monitored and treated by medical personnel on-site for non-SAEs. In the event of an SAE, emergency medical services (EMS) would be notified immediately and supportive care (e.g., StatKit® Emergency Medical Kits, HealthFirst) administered by medical staff as needed until the arrival of EMS.

Laboratory data (hematology, serum chemistry, urinalysis, and virology [human immunodeficiency virus (HIV) antibodies, HBsAg, anti-HBc (total), anti-HCV]) were collected at visit 1 (screening) and at visit 2 (day 1) after the final scintigraphic image was taken prior to dinner. Laboratory parameters, for which reference ranges were available, were categorized with respect to the reference ranges as high, low, normal, and missing.

Adverse events included any abnormal laboratory test results (hematology, clinical chemistry, or urinalysis) or other safety assessments (e.g., electrocardiograms, radiological scans, vital sign measurements), including those that worsened from baseline, and were considered clinically significant in the medical and scientific judgment of the investigator (i.e., not related to the progression of underlying disease); an exacerbation of a chronic or intermittent pre-existing condition including either an increase in frequency and/or intensity of the condition; new conditions detected or diagnosed after study treatment administration even though they might have been present before the start of the study; signs, symptoms, or the clinical sequelae of a suspected drug-drug interaction; and signs, symptoms, or the clinical sequelae of a suspected overdose of either study treatment or a concomitant medication. Recording of AEs was conducted at both visits 1 and 2 and until 5 days following the last administration of the powder or caplet formulation.

### Statistics

For the analyses, the safety population consisted of all participants who received a radiolabeled treatment irrespective of whether they were included in the scintigraphy analysis. The scintigraphy analysis population included all participants who received radiolabeled treatment, did not vomit within 60 min after study drug administration, had sufficient data to determine the time to onset and completion of GE, and had no major protocol deviations. Scintigraphy images were corrected for radioactive decay and background radiation. The scintigraphy variables were summarized by treatment group using descriptive statistics. There were no formal clinical hypotheses tested in this study; therefore, a formal sample size calculation was not performed although it was planned to screen approximately 42 subjects to allow for at least 12 evaluable subjects per treatment group, a number sufficient to provide descriptive statistics. The statistical analysis software used was the SAS software version 9.4 or higher (SAS Institute, Cary, NC).

## Results

### Participants

**Fig. 2:**
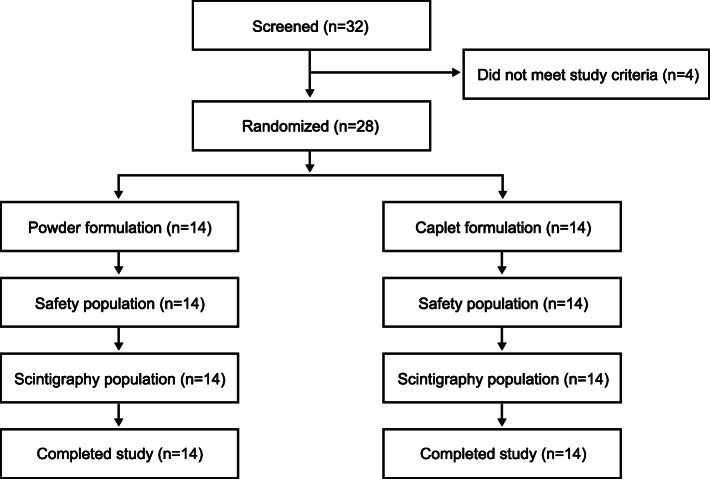
Participant disposition

A total of 32 participants were screened, 28 of whom were randomized 1:1 to receive the powder or caplet formulation and successfully completed the study (Fig. 1). The CONSORT flow diagram is presented in Fig. 2, and the CONSORT checklist appears in Fig. S1. The remaining 4 participants were excluded because they did not meet the study criteria. All participants were male with a mean ± SD age of 28.4 ± 7.0 years, and basic demographic characteristics were well matched between the powder and caplet formulation groups (Table 1).

**Table 1:** Demographic and baseline characteristics (safety population)

	Powder formulation (***n*** = 14)	Caplet formulation (***n*** = 14)	Overall (***N*** = 28)
**Sex,** ***n*** **(%)**			
Male	14 (100)	14 (100)	28 (100)
**Age, years**			
Mean ± SD	28.0 ± 8.74	28.8 ± 5.19	28.4 ± 7.06
Median (min, max)	23.5 (21, 45)	28.0 (21, 39)	26.5 (21, 45)
**Height, cm**			
Mean ± SD	183.1 ± 6.53	180.6 ± 7.00	181.9 ± 6.76
Median (min, max)	183.0 (170, 193)	180.0 (168, 196)	181.0 (168, 196)

*SD* standard deviation

### Primary GE endpoints

**Fig. 3:**
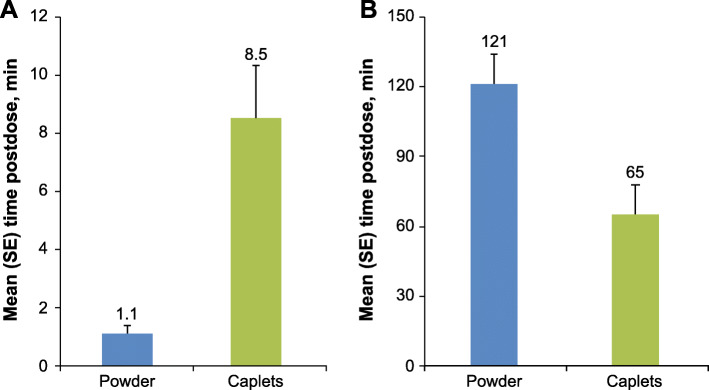
Mean time to GE onset (**A**) and completion (**B**) post-ingestion (scintigraphy analysis population). GE, gastric emptying; SE, standard error

External gamma scintigraphy demonstrated that the powder formulation in solution had a more rapid mean time to GE onset than the caplet formulation. The mean ± SE GE onset time for the powder was 1.1 ± 0.3 min and 8.5 ± 1.8 min for the caplet formulation (Fig. 3A). The mean ± SE time to GE completion was 121 ± 13 min for the powder formulation and 65 ± 13 min for the caplet formulation (Fig. 3B).

### Other scintigraphy endpoints

**Fig. 4:**
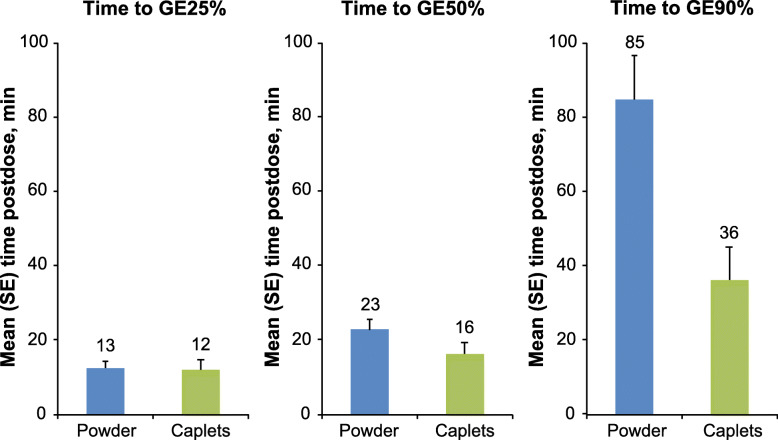
Mean time to 25%, 50%, and 90% GE post-ingestion (scintigraphy analysis population). GE, gastric emptying; SE, standard error

The powder (13 ± 1.8 min) and caplet (12 ± 2.4 min) formulations had similar mean ± SE times to GE25%. The caplet formulation had earlier mean GE50% and GE90% times compared with the powder formulation. Specifically, the mean ± SE GE50% values were 23 ± 3.0 min and 16 ± 3.2 min for the powder and caplet formulations, respectively, and the mean ± SE GE90% values were 85 ± 12 min and 36 ± 9.0 min, respectively (Fig. 4).

**Fig. 5:**
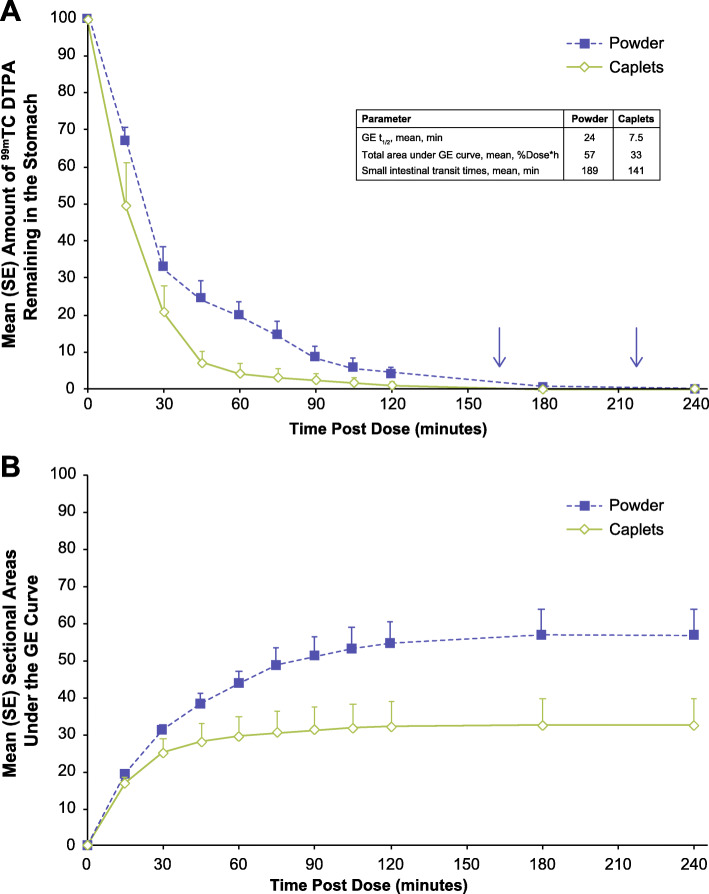
Mean post-ingestion ^99m^Tc DTPA stomach levels over time (scintigraphy analysis population). **A** Amount remaining. **B** Sectional areas under the GE curve. ^99m^Tc DTPA, technetium-99m diethylenetriaminepentaacetic acid; GE, gastric emptying; SE, standard error; t½, terminal half-life

Caplets had a faster GE half-life (t_1/2_), reduced gastric exposure (area under the GE curve), and faster transit time through the small intestine versus the powder formulation (Fig. 5A). The mean ± SE GE AUC (via the percentage of radioactivity present in the stomach at a given time point) was 57 ± 7.1 %dose × hour and 33 ± 7.1 %dose × hour for the powder and caplet formulations, respectively (Fig. 5B). The mean ± SE GE half-lives for the powder and caplet formulations were 24 ± 3.4 min and 7.5 ± 2.4 min, respectively. Finally, the mean ± SE small intestinal transit times for the powder and caplet formulations were 189 ± 12 min and 141 ± 16 min, respectively.

### Safety

There were no deaths or serious TEAEs reported during the study, and none of the participants reported any TEAE that led to discontinuation of study product or withdrawal from the study (Table 2). A total of 4 treatment-emergent AEs (TEAEs) were reported in 3 participants. One (7.1%) participant in the powder formulation group reported 1 event of treatment-related fatigue and 2 (14.3%) in the caplet formulation group reported 3 TEAEs (treatment-related increased alanine aminotransferase [ALT] in 1 participant and diarrhea and nausea in 1 participant). The events of fatigue and increased ALT were mild in intensity, while the events of diarrhea and nausea were of severe intensity.

**Table 2:** Safety summary (safety population)

Characteristic	Powder formulation (***n*** = 14)	Caplet formulation (***n*** = 14)
Participants with ≥ TEAE, *n* (%)	1 (7.1)	2 (14.3)
TEAEs, *n*	1	3
Discontinuations due to TEAEs, *n* **(**%)	0	0
Treatment-related TEAEs, *n* (%)	1 (7.1)	1 (7.1)
SAEs, *n* (%)	0	0
Deaths, *n* (%)	0	0
TEAEs by preferred term, *n* (%)		
Diarrhea	0	1 (7.1)
Fatigue	1 (7.1)	0
Increased ALT level	0	1 (7.1)
Nausea	0	1 (7.1)

*ALT* alanine aminotransferase, *SAE* serious adverse event, *TEAE* treatment-emergent adverse event

All AEs were resolved by study completion, except increased ALT. This AE was ongoing at the last report; the participant completed the study but did not return for additional follow-up, so the resolution was not fully documented.

## Discussion

The common cold, a self-limiting viral-induced respiratory infection, is the most frequently experienced infection among humans. Although to date there is no cure, numerous therapeutic products are available to provide palliative relief, reduce symptom severity, and prevent or mitigate the detrimental impacts of illness on quality of life. Oral administration is the most convenient and common means of drug delivery: dosage forms include tablets, capsules, powders, syrups, and sachets. Rapid GE is a key approach to reducing the delay between drug ingestion and the onset of symptom control.

This is the first study to characterize the GE properties of two distinct oral cold remedy formulations in healthy male adults, including time of onset and completion of GE and small intestinal transit time. Time to GE onset was more rapid with the powder in solution than the oral caplets (1.1 min vs 8.5 min, respectively), and starting at approximately 8 min post-dose, the 100% GE rate was faster for caplets, resulting in an earlier GE completion time for the caplet versus the powder formulation (65 min vs 121 min). As expected, no serious safety events were observed with either treatment.

The powder formulation reconstituted in hot water had a later GE completion than the caplet. The reason for this observation is unclear, but the prolonged GE of the powder in solution may have occurred due to the higher volume of dispersed radioactivity in the solution. It is also possible that, because of the high sugar content present in the powder formulation, the liquid formulation may have had a higher caloric density or greater osmolality, both of which are identified as regulators of GE rates [21]. Moreover, emptying of the intact caplet from the stomach upon dosing prior to disintegration resulted in faster onset and rapid rate of GE in 1 participant, and the location of caplets near the antrum or pylorus after dosing in approximately two-thirds of participants receiving the caplet formulation resulted in faster caplet disintegration followed by early-onset and a rapid rate of GE. These findings provide additional means by which the more rapid GE of the caplet versus the powder formulation can be explained.

Treatments that most effectively reduce cold symptoms have the potential to ease the economic burden associated with the common cold [1–3]. Hot drink remedies are associated with greater subject comfort and directly provide active ingredients in solution [7], which may result in the active ingredients reaching the bloodstream faster and being bioavailable more quickly compared with tablet formulations [6]. Prior research demonstrated that an unmedicated hot fruit drink can provide subjective relief from runny nose, cough, sneezing, sore throat, chilliness, and tiredness, whereas the same drink at room temperature provided relief for only half of these symptoms [7]. Hot drinks likely provide cold symptom relief by a placebo effect or may impart physiological effects such as salivation and airway mucus secretions to lubricate and soothe the upper airways, and/or stimulation to the trigeminal nerves that supply the oral and nasal cavities [7]. Any such benefit of a hot-temperature drink may be rapidly abolished once ingested, as the stomach quickly equilibrates the temperature of the ingested beverage to the internal body temperature, making it unlikely that significant amounts of markedly hot or cold liquids reach the duodenum [21].

Despite the value of this study in examining well-matched treatment groups and a robust assessment technique to evaluate the transport of the formulations through the GI tract, there were several constraints imposed by the study design. First, all participants in this study were male, as the study was designed to exclude females based on potential differences in GE during menstruation. Although the exclusion of females was important to control experimental variables to potentially elucidate GE differences, it limits translation of the findings to a broader population. Second, this study was small in size and descriptive, as no formal hypothesis testing or statistical significance was performed; moreover, at the time of our study, no prior data were available to aid in defining a statistical hypothesis. Third, this study lacked pharmacokinetic and pharmacodynamics data to correlate the timing of GE with the rate and extent of absorption of the active ingredients. Fourth, none of the participants, investigators, or analysts were blinded to the treatment. However, as the scintigraphy outcomes are not subjective, treatment knowledge would be very unlikely to result in bias. Finally, the assessments conducted were of marketed products; therefore, this was not an excipient or formulation study that could allow for formula manipulations to ascertain the precise mechanism(s) responsible for the disparity between GE times of the caplet and powder formulations.

## Conclusion

This is the first clinical study to characterize the GI transit of two powder and caplet formulations of commercially available cold symptom relief products. Time of onset, completion of GE, and small intestinal transit time were successfully assessed and quantified using gamma scintigraphy. The results suggested that the powder formulation in solution had a more rapid mean time to GE onset, but slower time to GE completion, than the caplet formulation in healthy male adults. Although the powder formulation in solution had a slower GE rate than the caplet, no correlation with the pharmacokinetics or pharmacodynamics profiles is available, or their potential correlation with cold symptom relief. There were no serious safety events observed with either treatment in this small, descriptive, exploratory study. Future hypothesis-driven trials of the GI transit of these formulations in a broader population of subjects affected by the common cold are warranted to establish the clinical relevance of our findings.

## Supplementary Information

**Additional file 1 Fig. S1.** CONSORT 2010 checklist of information to include when reporting a randomised trial*.

## Data Availability

Anonymized individual participant data and study documents can be requested for further research from www.clinicalstudydatarequest.com.

## Abbreviations

^99m^Tc DTPA: Diethylenetriaminepentaacetic acid
AE: Adverse event
ALT: Alanine aminotransferase
AUC: Area under the gastric emptying curve
EMS: Emergency medical services
GCP: Good Clinical Practice
GE: Gastric emptying
GE25%: 25% of drug remaining in the stomach
GE50%: 50% of drug remaining in the stomach
GE90%: 90% of drug remaining in the stomach
GI: Gastrointestinal
ICH: International Council for Harmonisation
IRB: Institutional review board
SAE: Serious adverse event
SD: Standard deviation
SE: Standard error
t_1/2_: Terminal half-life
TEAE: Treatment-emergent adverse event
